# Ticks (Acari: Ixodoidea) in China: Geographical distribution, host diversity, and specificity

**DOI:** 10.1002/arch.21544

**Published:** 2019-03-11

**Authors:** Yan‐Kai Zhang, Xiao‐Yu Zhang, Jing‐Ze Liu

**Affiliations:** ^1^ Hebei Key Laboratory of Animal Physiology, Biochemistry and Molecular Biology, College of Life Sciences Hebei Normal University Shijiazhuang Hebei China

**Keywords:** China, geographical distribution, host diversity, host specificity, ticks

## Abstract

Ticks are obligate blood‐sucking ectoparasites, which not only directly damage through bites but also transmit many pathogens. China has a high diversity of tick species, 125 species have been reported, including 111 hard tick and 14 soft tick species. Many of the ticks are important vectors of pathogens, resulting in zoonoses. The dynamics of ticks are affected by both the host and habitat environment. However, systematic studies on the geographical distribution, host diversity, and specificity of ticks are limited in China. To achieve this goal, the relevant available data were summarized and analyzed in this study. Ticks are distributed in all parts of China and Xinjiang has the most records of ticks. The distribution of ticks in adjacent areas is similar, indicating that the habitat environment affects their distribution. Most ticks are widely distributed, whereas some species are endemic to their distributed regions. Ticks are parasitic on mammals, birds, and reptiles, of which mammals are the main host species. Overall, most ticks parasitize different hosts, only a few ticks have strict host specificity, such as ticks that are specifically parasitic on reptiles and bats. In addition, environmental changes and control efforts also influence the dynamics of ticks. These results can better reveal tick biological traits and are valuable for tick control.

## Graphical abstract

Ticks distribute in whole regions of China, and their distributions are mainly influenced by habitat environment. Most ticks feed on different hosts, only a few ticks have strict host specificity.

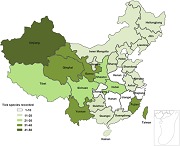

## 1 INTRODUCTION

Ticks are obligate blood‐sucking arthropods found throughout most regions of the world. Ticks infest every class of terrestrial vertebrates, including mammals, birds, reptiles, and even amphibians (Sonenshine & Roe, 2013). Ticks are important to humans through the direct effects of their feeding and as vectors for various agents of disease in both man and livestock (de la Fuente, Estrada‐Pena, Venzal, Kocan, & Sonenshine, 2008). More than 16 specific tick‐borne (or tick‐caused) diseases of humans and more than 19 tick‐borne diseases of livestock and companion animals have been described to date (Nicholson, Sonenshine, Lane, & Uilenberg, 2009). The frequencies of several tick‐borne diseases and their geographical distribution are increasing, owing in part to climatic changes (Estrada‐Peña, Ayllón, & De La Fuente, 2012; Gray, Dautel, Estrada‐Peña, Kahl, & Lindgren, 2009). For example, the incidence of Lyme disease (LD), a well‐known tick‐borne disease, dramatically increased and spread to all parts of the United States in recent years (https://www.cdc.gov/lyme/index.html). In addition, tick infestation causes economic losses due to body weight or milk production loss or treatment cost used for its prevention and control. For example, at least 80% of the world's cattle population are at risk from tick‐borne diseases (Ghosh, Azhahianambi, & Yadav, 2007). Ticks cause a huge loss to cattle milk production conjugated with nutritional status and a poor management pattern in Ethiopia (Duguma, Kechero, & Janssens, 2012). Over 900 tick species have been identified to date globally, and they mostly belong to two families, the Ixodidae or hard ticks, and the Argasidae or soft ticks (Barker & Murrell, 2004; Guglielmone et al., 2010). Furthermore, hard ticks are divided into Prostriata (genus *Ixodes*) and Metastriata (remaining genera). In nature, most hard ticks are non‐nidicolous, shelter in leaf litter, duff, rotting vegetation, or are buried in sand and under gravel and stones. Almost all of the non‐nidicolous ones are species of the Metastriata subgroup of the Ixodidae. Most Argasidae and many Prostriata ticks are nidicolous, living in nests, burrows, caves, or other shelters used by their hosts or hiding close by. There are four stages, namely egg, larva, nymph, and adult in the life cycle of ticks. Ticks spend most of their life cycle off the host, exposed to these environmental conditions, and they will complete many important biological processes during the nonparasitic phase, such as moulting of engorged larvae and nymphs, oviposition of engorged females, incubation of eggs and host seeking. For larvae, nymphs, and adults, they need to feed or mate on the host at certain periods. The engorged ones detach from the host to seek shelter in a suitable microenvironment, where they can digest its meal and molt to the next stage. Depending on whether larvae and nymphs molt to the nymph or adult stage, respectively, off or on the host, hard ticks are classified as 3‐, 2‐, or 1‐host ticks, and most of them have a 3‐host life cycle, taking at least a year to complete their life cycle. However, life cycles of the soft ticks are more variable than those of hard ticks. For instance, soft ticks have multiple nymph stages that require a blood meal, and soft ticks tend to feed rapidly, whereas hard ticks tend to complete feeding processes that last days (Sonenshine & Roe, 2013).

Ticks have evolved several different strategies to facilitate their survival, while waiting for vertebrate hosts. In non‐nidicolous species, the ticks crawl up emergent vegetation, where they wait for a passing host (the ambush strategy), and the questing periods might last many weeks or even months. Many tick species are able to survive for long periods because they can minimize evaporative water loss. Some ticks use the hunter strategy in host seeking, and they are attracted by CO_2,_ ammonia, and other odorants, emerge from their shelters and walk or run toward their hosts. Nidicolous ticks exhibit very different behavioral patterns than those of non‐nidicolous species. They shelter in the nests or burrows of their preferred hosts (or close by). There they are exposed to greater humidity and less extreme environmental conditions than their non‐nidicolous relatives. These ticks rarely, if ever, are found questing outside of the host's dwelling. All stages of most of the nidicolous ticks feed on the same type of host. The nidicolous lifestyle often leads to host specificity, as it does to some degree in many species of Ixodidae ticks (J. Z. Liu & Yang, 2013; Sonenshine & Roe, 2013). Obviously, the life cycle and population dynamics of ticks are influenced by two factors, host seeking efficiency and host availability. The former factor is often associated with the population density, distribution pattern and activity periods of the host and the environmental conditions in tick seeking regions. Host availability refers to whether the host can be used or preferred by ticks. Previous studies have conformed two contrasting hypotheses concerning tick–host associations: Ticks select certain hosts in a given environment, and ticks select certain environments and feed on any available host within these (Cumming, 1998; Hoogstraal & Aeschlimann, 1982; Klompen, Black, Keirans, & Oliver, 1996; Nava & Guglielmone, 2013). In a broad sense, the host specificity of ticks is quantified as the number of host species, or host range. However, meta‐surveys found a significant correlation between host range and sampling effort and suggested that ticks are habitat rather than host specialists, thus simply counting the number of hosts used by ticks cannot fully reflect the host specificity degree (Cumming, 1998; Nava & Guglielmone, 2013). At the scale of the global geographic distribution of a species, ticks tend to be host generalists, and the ecological similarity of the host environment may be more important than host phylogenetic similarities in determining the ticks’ host range. At a more local scale, host specialization seems to be the norm in ticks. In addition, the host range of ticks is often related to tick‐borne pathogen circulation and disease risk (Mccoy, Léger, & Dietrich, 2013). Taken together, evaluating the host range of a particular tick species is more informative in understanding tick ecology and evolution.

China has a relatively high tick species diversity, and over 120 species (about 13% of world species) have been described across its geography and infesting different hosts (Bush & Robbins, 2012; Chao, Hsieh, & Shih, 2013; Chao, Lu, Lin, & Shih, 2017; Z. Chen et al., 2010; Deng & Jiang, 1991; Duan, 2013; T. Guo, Sun, Xu, & Durden, 2017; Y. Guo, Sun, & Xu, 2016; W. Li, Sun, Zhang, & Xu, 2015; Wen & Chen, 2016; Yin & Luo, 2007; X. Yu, Ye, & Gong, 1997). Owing to the medical and economic importance of ticks, increasing studies have paid more attention to tick biology (Z. Chen, Yu, Yang, Zheng, & Liu, 2009; J. Li et al., 2018; J. Liu, Liu, Zhang, Yang, & Gao, 2005; Ma et al., 2016), tick ecology (T. Wang et al., 2017; Zheng, Yu, Zhou, Yang, & Liu, 2012), and tick‐borne disease (Chu et al., 2008; Fang et al., 2015; Wu, Na, Wei, Zhu, & Peng, 2013; Z. Yu et al., 2015), especially reports of the new emergence of tick‐borne pathogens in recent years (H. Li et al., 2015; Y. Z. Zhang & Xu, 2016). These extensive efforts provide valuable information for tick control. However, the studies with an emphasis on geographical distribution, host diversity, and specificity of ticks are limited to China. Many studies only briefly recorded the distribution and host of tick species at a local scale (G. P. Liu, Ren, He, Wang, & Yang, 2008; Y. Z. Wang et al., 2015; Y. S. Yang et al., 2008). In this present study, the geographical distribution, host diversity, and specificity of ticks in China will be discussed based on available data.

## 2 TICK SPECIES RECORDED IN CHINA

The data on tick species distributed in China are mainly obtained from published literature (Z. Chen et al., 2010; Deng & Jiang, 1991; Duan, 2013; T. Guo et al., 2017; Y. Guo et al., 2016; W. Li et al., 2015; Wen & Chen, 2016; X. Yu et al., 1997). Wen and Chen (2016) also examined and revised the valid species names of the Argasidae ticks of the world. A total of 125 tick species have been recorded and described in the area of study. Based on these data, the detailed information on the valid species names and distributions are presented in Table 1. As shown, the Ixodidae family comprises 111 species from seven genera, *Amblyomma* (eight species), *Anomalohimalaya* (two species), *Dermacentor* (14 species), *Haemaphysalis* (43 species), *Hyalomma* (seven species), *Ixodes* (29 species), and *Rhipicephalus* (eight species), and the Argasidae family comprises 14 species from two genera, *Argas* (10 species), and *Ornithodoros* (four species). Considering that some new tick species and new recorded tick species were recently reported (T. Guo et al., 2017; Y. Guo et al., 2016; H. Li et al., 2015; Wen & Chen, 2016), and China has a vast territory and various landscape types, the number of tick species in China will most likely increase in future study efforts.

**Table 1:** Geographical distribution and natural hosts of ticks in China

Tick tick species	Geographical distribution	Natural hosts
Hard ticks		
*Ixodes persulcatus* Schulze	Heilongjiang, Jilin, Liaoning, Shanxi, Xinjiang, Tibet	Livestock^a^, large mammals^a^, humans^a^, birds^b^, small mammals^b^
*I. sinensis* Teng	Fujian, Jiangxi, Hunan, Yunnan, Zhejiang, Anhui, Hubei	Cattle, buffaloes, sheep, leopard, mice, humans
*I. nuttallianus* Schulze	Yunnan, Sichuan, Qinghai, Tibet	Cattle, buffaloes, sheep, goats, dogs, deer
*I. hyatti* Clifford, Hoogstraal *et* Kohls	Hubei, Yunnan, Sichuan, Xinjiang, Zhejiang	Rodentia
*I. crenulatus* Koch	Shandong, Ningxia, Sichuan, Qinghai, Xinjiang, Tibet, Gansu, Inner Mongolia, Heilongjiang, Jilin, Liaoning	Cave dwelling mammals, dogs, birds
*I. arboricola* Schulze *et* Schlottke	Guangxi, Tibet, Xinjiang, Inner Mongolia	Birds
*I. moscharius* Teng	Tibet	*Moschus berezouskii*
*I. moschiferi* Nemenz	Sichuan, Qinghai, Tibet	*M. moschiferus*, *M. berezouskii*, *M. sifanicus*
*I. pomerantzevi* Serdjukova	Shandong, Hubei, Shanxi, Shaanxi, Sichuan, Gansu, Liaoning	Livestock, *Vulpes vulpes*, *Eutamias sibiricus*
*I. vespertilionis* Koch	Jiangsu, Fujian, Taiwan, Hubei, Shanxi, Guizhou, Yunnan, Sichuan, Liaoning, Inner Mongolia, Guangxi	Bats
*I. simplex* Nenmann	Jiangsu, Fujian, Taiwan, Guizhou, Shanghai	Bats
*I. tanuki* Saito	Gansu, Tibet	*Nyctereutes procyonoides*, *Mustela altaica*, *M. sibirica, Martes flavigula*
*I. myospalacis* Teng	Shaanxi, Ningxia, Qinghai, Gansu	*Myospalax fontanieri*
*I. kangdingensis*	Sichuan	*M. sibirica*
*I. kashmiricus* Pomerantzev	Tibet	Cattle^a^, small mammals^b^
*I. semenovi* Olenev	Tibet	Birds
*I. ovatus* Neumann	Anhui, Fujian, Taiwan, Guangxi, Hubei, Shaanxi, Ningxia, Guizhou, Yunnan, Sichuan, Qinghai, Tibet, Gansu	Livestock, humans, *Elaphodus cephalophus*, *Naemorhedus goral*, *M. berezovskii*, *M. chrysogaster*, *M. sibirica, Ailuropoda melanoleuca*
*I. acutitarsus* Karsch	Taiwan, Hubei, Yunnan, Tibet, Gansu	Cattle^a^, buffaloes^a^, goats^a^, dogs^a^, bears^a^, sylvatic pigs^a^, *Pseudois nayaur* a, *N. goral* a, *M. berezovskii* a, *Urocissa erythroryncha* a, Insectivora^b^, Rodentia^b^
*I. granulatus* Supino	Jiangsu, Fujian, Guangdong, Guangxi, Taiwan, Hainan, Hubei, Guizhou, Yunan, Sichuan, Tibet	Insectivora, Rodentia, Carnivora
*I. kuntzi* Hoogstraal *et* Kohls	Taiwan, Hubei, Xinjiang	Rodentia, *Sitta curopaea*
*I. spinicoxalis* Neumann	Fujian	Small mammals
*I. kazakstani* Olenev	Xinjiang	Livestock
*I. pavlovskyi* Pomerantzav	Xinjiang, Jilin	*Columba rupestris*
*I. apronophorus* Schulze,	Xinjiang	*Rattus norvegicus*
*I. frontalis* Panzer	Xinjiang	NR
*I. nipponensis* Kitaoka et Saito	Taiwan	Reptiles
*I. redikorzevi* Olenev	Xinjiang	*Vormela peregusna*, *Hemiechinus auritus*
*I. berlesei* Birula	Xinjiang	Livestock, wild mammals
*I. pavlovskyi* Pomerantzav	Shanxi, Jilin	Birds^a^, ^b^, hedgehogs^a^, small mammals^b^
*Haemaphysalis kitaokai* Hoogstraal	Hebei, Taiwan, Yunnan, Sichuan, Xinjiang, Gansu	Cattle, horses, deers
*H. aponommoides* Warburton	Fujian, Hainan, Tibet	Cattle, buffaloes, bears
*H. vietnamensis* Hoogstraal *et* Wilson	Fujian, Hunan, Guizhou, Yunnan	Cattle, buffaloes
*H. primitiva* Teng	Yunnan, Sichuan	NR
*H. warburtoni* Nuttall	Shaanxi, Sichuan, Tibet	Cattle, buffaloes, sheep
*H. danieli* Cerny *et* Hoogstraal	Tibet, Xinjiang, Gansu	Sheep, Rodentia
*H. tibetensis* Hoogstraal	Tibet, Xinjiang, Gansu	Dogs, sheep, cattle, buffaloes,
*H. garhwalensis* Dhanda *et* Bhat	Tibet	Sheep
*H. punctata* Canestrini *et* Fanzago	Shaaxi, Qinghai, Xinjiang, Gansu	Large mammals^a^, birds^a^, ^b^, small mammals^b^
*H. sulcata* Canestrini *et* Fanzago	Xinjiang	Livestock^a^, reptiles^b^
*H. nepalensis* Hoogstraal	Hubei, Tibet	Cattle, humans
*H. formosensis* Neumann	Fujian, Taiwan, Hainan, Yunnan	Sylvatic pigs, dogs
*H. aborensis* Warburton	Yunnan	Large mammals^a^, small mammals^b^, birds^b^
*H. concinna* Koch	Hebei,Anhui, Shanxi, Shaanxi, Ningxia, Xinjiang, Gansu, Inner Mongolia, Heilongjiang, Jilin, Liaoning	Large mammals^a^, humans^a^, small mammals^b^, birds^b^
*H. japonica* Warburton	Hebei, Hubei, Shanxi, Shaanxi, Ningxia, Qinghai, Xinjiang, Gansu, Inner Mongolia, Heilongjiang, Jilin, Liaoning	Large mammals^a^, humans^a^, Rodentia^b^, birds^b^
*H. campanulata* Warburton	Hebei, Shandong, Jiangsu, Fujian, Hubei, Shanxi, Ningxia, Sichuan, Inner Mongolia, Heilongjiang	Dogs, other mammals
*H. megaspinosa* Saito	Sichuan, Gansu	Cattle, buffaloes, *A*. *melanoleuca*
*H. qinghaiensis* Teng	Ningxia, Yunnan, Sichuan, Qinghai, Tibet, Gansu	Large mammals
*H. moschisuga* Teng	Yunnan, Sichuan, Qinghai, Tibet, Gansu	*M. berezovskii*, *Bos mutus*
*H. flava* Neumann	Jiangsu, Taiwan, Hubei, Guizhou, Yunan, Sichuan, Gansu	Large mammals
*H. goral* Hoogstraal	Zhejiang	*N. goral*
*H. sinensis* Zhang	Hubei, Shaanxi	Cattle, goats
*H. birmaniae* Supino	Taiwan, Guangxi, Yunnan	Artiodactyla
*H. wellingtoni* Nuttall *et* Warburton	Fujian, Yunnan	Poultry, birds, dogs, buffaloes
*H. montgomeryi* Nuttall	Guizhou, Yunnan, Sichuan, Tibet	Livestock
*H. longicornis* Neumann	Heibei, Shandong, Henan, Anhui, Jiangsu, Taiwan, Guangdong, Hunan, Hubei, Shanxi, Shaanxi, Guizhou, Sichuan, Qinghai, Tibet, Gansu, Heilongjiang, Jilin, Liaoning	Livestock, wild mammals, birds, humans
*H. bispinosa* Neumann	Anhui, Jiangsu, Fujian, Hunan, Hubei	Cattle
*H. mageshimaensis* Saito *et* Hoogstraal	Anhui, Fujian, Taiwan, Yunnan	Livestock, wild mammals, birds, humans
*H. hystricis* Supino	Jiangsu, Fujian, Taiwan, Guangdong, Hainan, Yunnan	Dogs, buffaloes, tigers, humans, sylvatic pigs, *Arctonyx collaris*, *Rusa unicolor*, *Muntiacus reevesi*, Hystricidae, *Martes flavigula*,
*H. yeni* Toumanoff	Fujian, Taiwan, Hainan, Hunan, Hubei, Yunnan	*Dogs*, *R. unicolor*, *Cuon alpinus*
*H. lagrangei* Larrousse	Hainan	*Muntiacus muntjak*, *M.* sp.
*H. spinigera* Neumann	Fujian, Guangdong, Hubei, Yunan	Cattle, tigers, bears, leopards, *R. unicolor*
*H. cornigera* Neumann	Fujian, Taiwan, Guangdong, Guangxi, Hainan, Yunan	Buffaloes, cattle
*H. anomaloceraea* Teng	Yunnan	NR
*H. verticalis* Ltagaki, Noda *et* Yamaguchi	Hebei, Hubei, Shanxi, Ningxia, Qinghai, Gansu, Inner Mongolia, Heilongjiang, Jilin, Liaoning	*Citellus dauricus*, *Procapra przewalskii*, cattle, dogs, small mammals
*H. doenitzi* Warburton *et* Nuttall	Fujian, Taiwan, Hainan, Yunnan	Birds, *Lepus sinensis*
*H. phasiana* Saito, Hoogstraal *et* Wassef	Jiangxi, Taiwan, Guizhou, Yunnan	Birds
*H. ornithophila* Hoogstraal *et* Kohls	Taiwan, Guangxi, Yunnan, Gansu	Birds, *M. sibirica*, *Pseudois nayaur*
*H. bandicota* Hoogstraal *et* Kohls	Taiwan	*Bandicota* sp.
*H. erinacei turanica* Pospelova‐Strom	Shanxi, Shaanxi, Ningxia, Xinjiang	*Rhombomys opimus*, *C. dauricus*, *Tamias sibiricus*, *M. eversmanii*
*H. asiatica* (Supino)	Fujian, Taiwan, Yunnan	Carnivora
*H. canestrinii* (Supino)	Fujian, Taiwan, Yunnan	Carnivora^a^, *Lepus peguensis* a, *Gallus gallus* b, small mammals^b^
*H. taiwana* Sugimato	Fujian, Taiwan, Guangdong, Guangxi, Yunnan	Sylvatic pigs, dogs
*Dermacentor reticulatus* (Fabricius)	Shanxi, Shaanxi, Xinjiang	Livestock^a^, wild mammals^a^, Rodentia^b^, Insectivora^b^, Lagomorpha^b^
*D. nuttalli* Olenev	Hebei, Anhui, Shaanxi, Ningxia, Qinghai, Xinjiang, Gansu, Inner Mongolia, Heilongjiang, Jilin, Liaoning	Livestock^a^, humans^a^, Rodentia^b^, small mammals^b^
*D. silvarum* Olenev	Hebei, Fujian, Shanxi, Shaanxi, Ningxia, Xinjiang, Gansu,Inner Mongolia, Heilongjiang, Jilin, Liaoning	Livestock^a^, wild mammals^a^, Rodentia^b^, small mammals^b^, birds^b^
*D. sinicus* Schulze	Hebei, Shandong, Shanxi, Ningxia, Xinjiang, Inner Mongolia, Heilongjiang, Jilin, Liaoning	Livestock^a^, wild mammals^a^, small mammals^b^
*D. niveus* Neumann	Shaanxi, Qinghai, Tibet, Xinjiang, Gansu, Inner Mongolia	Livestock^a^, large mammals^a^, small mammals^b^
*D. pavlovskyi* Olenev	Xinjiang	Livestock^a^, small mammals^b^
*D. montanus* Filippova *et* Panova	Xinjiang	Livestock^a^, small mammals^b^
*D. marginatus* (Sulzer)	Shanxi, Shaanxi, Xinjiang	Livestock^a^, Rodentia^b^
*D. abaensis* Teng	Sichuan, Qinghai, Tibet	Livestock^a^, large mammals^a^, small mammals^b^
*D. everestianus* Hirst	Qinghai, Tibet	Livestock^a^, large mammals^a^, Rodentia^b^
*D. auratus* Supino	Zhejiang, Jiangxi, Fujian, Taiwan, Guangdong, Hainan, Yunnan	Livestock^a^, large mammals^a^, Rodentia^b^, Primates^b^, Insectivorab, Carnivorab, birds^b^
*D. taiwanensis* Sugimoto	Fujian, Yunnan	Sylvatic pigs^a^, *A. melanoleuca* a, *Ursus thibetanus* a, Rodentia^b^, birds^b^
*D. atrosignarus* Neumann	Fujian	Sylvatic pigs
*D. raskemensis* Neumann	Xinjiang, Tibet	Sheep^a^, goats^a^, pikas^b^, hares^b^
*Amblyomma testudinarium* Koch	Zhejiang, Fujian, Taiwan, Guangdong, Guangxi, Hainan, Hunan, Yunan	Livestock, sylvatic pigs, tigers, humans, *R. unicolor*
*A. geoemygae* (Cantor)	Taiwan, Hunan	Tortoises
*A. javanense* (Supino)	Fujian, Guangdong, Guangxi, Hainan, Yunnan	Pangolins, reptiles
*A. helvolum* Koch	Taiwan, Hainan	Snakes
*A. cordiferum* Neumann	Taiwan	Reptiles
*A. varanense* (Supino)	Taiwan, Hainan	Reptiles
*A. crassipes* (Neumann)	Guangxi, Yunnan	Cattle, Pangolins, *Varanus salvator*
*A. pattoni* Neumann	Zhejiang, Guangdong	Snakes
*Hyalomma scupense* Schulze	Hebei, Henan, Shandong, Anhui, Hubei, Shanxi, Shaanxi, Ningxia, Qinghai, Xinjiang, Gansu, Inner Mongolia, Heilongjiang, Jilin, Liaoning	Livestock^a^, large mammals^a^, Rodentia^b^
*H. asiaticum asiaticum* Schulze *et* Schlottke	Shannxi, Ningxia, Qinghai, Xinjiang, Gansu, Inner Mongolia, Jilin	Livestock^a^, small mammals^a^, ^b^
*H. dromedarii* Koch	Qinghai, Xinjiang, Gansu	Camels, livestock, cave dwelling mammals
*H. anatolicum anatolicum* Koch	Xinjiang, Gansu	Livestock, wild mammals
*H. turanicum* Pomerantzev	Shanxi, Shaanxi, Inner Mongolia, Gansu, Ningxia, Xinjiang	Livestock^a^, birds^b^, rabbits^b^, hedgehogs^b^
*H. isaaci* Sharif	Fujian, Hainan, Yunnan, Sichuan	Camels, cattle, horses, sheep, donkeys, rabbits
*H. excavatum* Koch	Xinjiang	Livestock^a^, small mammals^a^, ^b^
*Rhipicephalus sanguineus* (Latreille)	Heibei,Shandong, Henan, Anhui, Jiangsu, Fujian, Taiwan, Guangdong, Guangxi, Hainan, Shanxi, Shaanxi, Ningxia, Guizhou, Yunnan, Tibet, Xinjiang, Gansu	Livestock^a^, small mammals^a^, ^b^
*R. turanicum* Pomerantzev	Shaanxi, Xinjiang	Livestock^a^, wild mammals^a^, Rodentia^b^, small mammals^b^
*R. pumilio* Schulze	Guangxi, Xinjiang, Inner Mongolia	Livestock^a^, wild mammals^a^, small mammals^b^
*R. bursa* Canestrini *et* Fanzago	Anhui, Xinjiang	Livestock, large mammals, rabbits
*R. haemaphysaloides haemaphysaloides* Supino	Hebei, Anhui, Zhejiang, Jiangsu, Fujian, Taiwan, Hainan, Guangdong, Guangxi, Hunan, Hubei, Guizhou, Yunnan, Tibet	Livestock^a^, wild mammals^a^, ^b^
*R. microplus* Canestrini	Hebei, Shandong, Henan, Anhui, Jiangsu, Zhejiang, Jiangxi, Fujian, Taiwan, Guangdong, Guangxi, Hainan, Hunan, Hubei, Shanxi, Shannxi, Guizhou, Yunnan, Sichuan, Tibet, Xinjiang, Gansu, Liaoning	Livestock, wild mammals
*R. rossicus* Yakimov et Kol‐Yakimova	Xinjiang	Livestock
*R. schulzei* Olenev	Xinjiang	*Spermophilus erythrogenys*
*Anomalohimalaya lama* Hoogstraal, Kaiser *et* Mitchell	Tibet	*Cricetulus kamensis*
*A. cricetuli* Teng *et* Huang	Xinjiang	*Cricetulus migratorius*
Soft ticks		
*Argas assimilis* Teng *et* Song	Jiangxi	*Cecropis daurica*
*A. beijingensis* Teng	Hebei, Inner Mongolia	Poultry, birds, humans
*A. japonicus* Yamaguti, Clifford *et* Tipton	Hebei, Jilin, Liaoning	Birds
*A. persicus* Oken	Hebei,Shandong, Anhui, Jiangsu, Fujian, Taiwan, Hubei, Shanxi, Shaanxi, Sichuan, Qinghai, Xinjiang, Gansu, Inner Mongolia, Heilongjiang, Jilin, Liaoning	Poultry, birds, humans
*A. robertsi* Hoogstraal, Kaiser *et* Kohls	Taiwan	NR
*A. vulgaris* Filippova	Hebei, Shandong, Shaanxi, Ningxia, Xinjiang, Gansu, Inner Mongolia	Livestock
*A. reflexus* (Fabricius)	Inner Mongolia, Hebei, Beijing, Ningxia, Shaanxi, Gansu, Xinjiang, Shandong	Pigeons
*A. pusillus* Kohls	Taiwan	Livestock
*A. sinensis* Jeu *et* Zhu	Chongqing	*Pipistrellus abramus*
*A. vespertilionis* (Latreille)	Hebei, Shandong, Xinjiang, Jiangsu, Hunan, Taiwan, Gungdong, Guangxi, Yunnan	Bats
*Ornithodoros lahorensis* Neumann	Xinjiang, Gansu	Livestock
*O. capensis* Neumann	Taiwan	Seabirds
*O. papillipes* (Birula)	Shanxi, Xinjiang	Livestock, small mammals, poultry, humans
*O. tartakovskyi* Olenev	Shaanxi, Xinjiang	Wild mice, humans

a Adult ticks.

b Immature ticks.

## 3 GEOGRAPHICAL DISTRIBUTION OF TICKS IN CHINA

**Figure 1:**
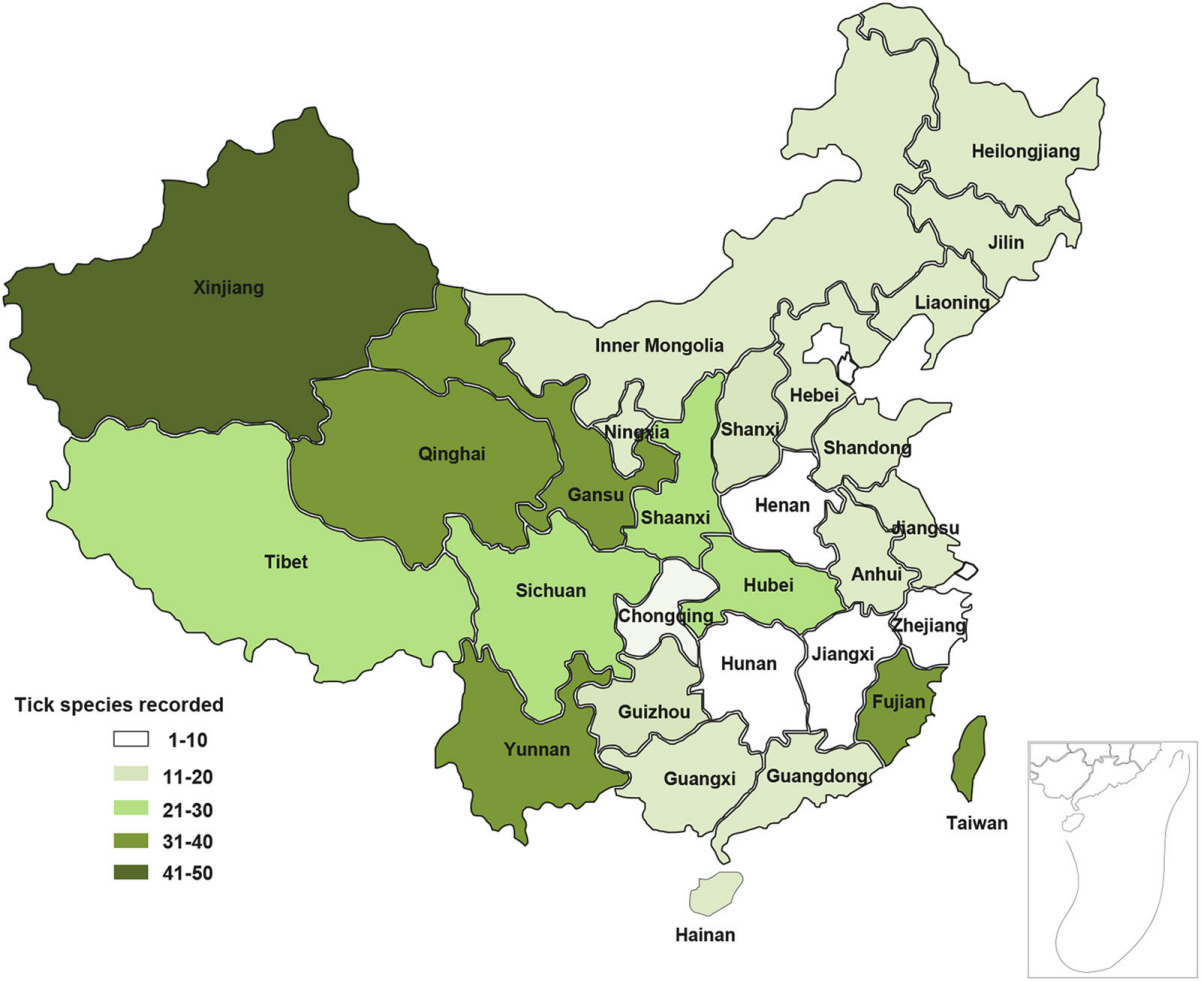
Tick species recorded in each province in China

In China, Deng and Jiang (1991) have revealed that tick species of *Anomalohimalaya* genus only occur in northern regions, whereas *Amblyomma* species are endemic in southern regions. Most of *Dermacentor* and *Hyalomma* species are mostly distributed in northern regions and their distributions in southern regions are scanty. Tick species of *Haemaphysalis*, *Ixodes,* and *Rhipicephalus* genera are widely distributed, but *Haemaphysalis* species are more frequent in the south and the other two genera are more common in the north. In a later study, Z. Chen et al. (2010) also described the geographical distribution of 117 tick species recorded in China and found that distribution of ticks appeared as spots or belts. In detail, about 70% tick species are endemic and exhibit spot distribution, they only occur in a single province or adjacent regions. These species have adapted to the distinct ecological conditions of their distributed regions. Although the universal tick species can cope with various ecological conditions and show belt distribution, these species account for about 30% of the population and many of them are the dominant species in China. For example, *Haemaphysalis longicornis*, *Hyalomma scupense*, and *Rhipicephalus microplus* distribute in most regions of China. In addition, there are many studies that focused on the geographical distribution of ticks at the province level (G. P. Liu et al., 2008; Y. Z. Wang et al., 2015; Y. S. Yang et al., 2008; Yu et al., 1997). Here, these data are reviewed and summarized, detailed information is present in Table 1, and the numbers of tick species recorded of each province are shown in Figure 1. As described in earlier studies, the geographical distributions of ticks are not even and variable among geographical regions. From an ecological perspective, the distributions of endemic and universal species are dependent on their adaption plasticity, when novel hosts occur or climate change within their habitat. Other factors that influence the distribution of ticks are the distributions and densities of available hosts, human activity, and tick control efforts.

Without consider the sampling bias, as shown in Figure 1, Xinjiang has the most tick species records (49 species), followed by Qinghai, Gansu, Yunnan, Fujian, and Taiwan, more than 30 species are found in these provinces. The provinces that have more than 20 species comprise Tibet, Sichuan, Shaanxi, and Hubei. Most of the remaining provinces have more than 10 species. It is worth noting that tick species records in Henan, Jiangxi, Hunan, Zhejiang, and Chongqing are relatively less, and tick species records in Beijing and Tianjin are lacking. Ecological differences among these regions mainly result in differences in tick species records. For example, Xinjiang has different ecological types from inland areas and its livestock and wildlife are rich, so that many endemic tick species can survive in this region. Similarly, some endemic tick species are found in Tibet that is located in the Qinghai‐Tibetan Plateau. Obviously, tick species records are strongly correlated with sampling and investigation efforts, ticks with great medical and agricultural importance receive more attention. In such cases, the tick species diversity of each province cannot be simply evaluated based on these records and more investigations of tick distribution are required, especially in areas where low numbers of tick species have been reported.

## 4 HOST DIVERSITY OF TICKS IN CHINA

There are differences in host distribution and diversity across tick distributed areas. In China, ticks can infest many vertebrates, including mammals, birds and reptiles, although their performances on these hosts remain largely unknown. X. J. Yang, Chen, Yang, and Liu (2008) have counted that about 130 animal species of 20 orders can be hosted by ticks in China. Of the 125 tick species examined in this study, nine species are from reptiles, 31 from birds, and 112 from mammals (Table 1). Furthermore, reptiles comprise lizards, snakes, and tortoises. Birds infested by ticks are mostly poultry, pigeons and wild birds. Like other studies, mammals are the main hosts of ticks, including Artiodactyla, Carnivora, Chiroptera, Insectivora, Lagomorpha, Perissodactyla, Pholidota, Primates, and Rodentia. Among the mammal hosts, most tick species are found on Artiodactyla and Rodentia, with 80 and 42 species of ticks respectively. In addition, many ticks are found on domestic animals, including cattle, buffaloes, sheep, goats, rabbits, pigs, dogs, horses, and donkeys, which are marked as livestock in this study. These ticks will cause host body weight or milk production loss and act as reservoirs of diseases, thus they receive much attention (Yin & Luo, 2007; Z. Yu et al., 2015). Some tick species can infest different classes of animals. For example, larvae and nymph ticks of *Ixodes persulcatus* are found on birds and small mammals. Cattle, pangolins and lizards are hosts of *Amblyomma crassipes*. These ticks that have wide host range may have adapted to different host types, including host habitats, host immunity, and so on. A series of experimental studies on the life cycle or reproductive barriers of ticks have also reinforced the hypothesis. In detail, under laboratory conditions, many ticks exhibited physiological plasticity as they can feed successfully using novel hosts not related to the natural hosts (Z. Chen et al., 2009; Faccini, Cardoso, Onofrio, Labruna, & Barros‐Battesti, 2010; Labruna, Soares, Martins, Soares, & Cabrera, 2011; J. Liu et al., 2005; Ma et al., 2013; Olegário, Gerardi, Tsuruta, & Szabó, 2011; Pinter, Dias, Gennari, & Labruna, 2004). Meanwhile, these findings raise the possibility that the host diversity of ticks is undervalued, and various hosts can be used by ticks. Assessment of the host diversity of ticks is strongly affected by sampling bias and data record anomalies. In such cases, the influences of the two factors should be paid more attention to in investigating the host diversity of ticks.

## 5 HOST SPECIFICITY OF TICKS IN CHINA

All tick species are obligate parasites, and their life cycles mainly depend on available food meals from suitable vertebrate hosts. Some tick species accept a wide variety of host species. For instance, *Ixodes scapularis* feed on lizards, birds, small mammals, and larger hosts like sheep and humans. Other tick species are more selective, and some are extremely demanding and feed primarily, or solely, on one host species (e.g., *Argas* ticks that feed only on bats, or *R. microplus* that feed only on large ruminants). There are some debates about host specificity in ticks. Ticks have traditionally been considered as relatively host‐specific, and their geographical distribution can be determined by that of their hosts (Hoogstraal & Aeschlimann, 1982). However, Klompen et al. (1996) concluded that adaptation to a particular habitat is more relevant for tick distributions than adaptation to a particular host. In a later study, Cumming (1998) suggested that both host specificity and ecological specificity could be important in determining tick distributional ranges and their evolution and that it depends on each particular tick species. A meta‐analysis of host specificity in Neotropical hard ticks revealed that strict host specificity is not common among these ticks and suggested that the influence of tick ecology and evolution of habitat specificity, tick generation time, phenology, time spent off the host, and the type of life cycle could be more important than host species (Nava & Guglielmone, 2013). Although strict host specificity is not common in ticks in these studies, many ticks exhibited host specificity to varying degrees. Analyzing tick–host specificity at different taxonomic levels of both ticks and their hosts, Cumming (1998) revealed that most of the well‐collected genera of African ticks are found mainly on mammals, with *Aponomma* specializing on squamate reptiles, *Carios* on bats and *Argas* on birds. Furthermore, host preference is more obvious at a specific level, and at least 39 tick species exhibit specialization to a taxonomic level below class. Field observations suggest that even broad host generalists tend to feed on only a few main hosts locally, with these hosts changing across different areas of the distribution (Balashov, 2010). Significant patterns of local host‐associated genetic structure have been observed in several tick species (McCoy et al., 2013). In these cases, ticks have adapted to different environmental conditions and thus preferred different hosts depending on availability in a particular area.

Studies of host specificity of ticks in China are relatively limited (Deng & Jiang, 1991). In this study, general patterns of tick–host specificity are described based on available data, although sometimes recorded data are incomplete. Some tick species were only found on a single host species, in such case, whether or not they have strict host specificity is not determined. Additionally, three tick species, *Ixodes frontalis*, *Haemaphysalis primitive*, and *Argas robertsi*, had no host records.

In general, hosts that can be used by ticks comprise reptiles, birds, and mammals. Among these tick species, nine species feed on reptiles, and five are specific for reptiles. Seven of the nine reptile‐feeding ticks belong to *Amblyomma* genus (Table 1). Most of them are distributed in several adjacent provinces in southern regions, which have a similar ecological environment. A particular case is *Haemaphysalis sulcate*, which is reported from Xinjiang and its immature ticks feed on reptiles for survival. Birds serve as natural hosts for 31 tick species, including *Ixodes*, *Haemaphysalis*, *Dermacentor*, *Hyalomma*, *Argas,* and *Ornithodoros* genera (Table 1). Among them, eight species are strictly parasites of birds and 23 are occasionally found on birds or their immature ticks parasitize birds. Ticks of *Argas* genus generally use birds as their natural hosts. None of the *Rhipicephalus* and *Amblyomma* species are specific parasites of birds. Adult *Dermacentor* species never feed on birds; immatures rarely do so. These findings are consistent with previous studies (Cumming, 1998; Hoogstraal & Aeschlimann, 1982). Mammals serve as hosts for more tick species than do birds and reptiles, and at least one species of each tick genus utilizes mammals as hosts (Table 1). Artiodactyla are hosts for 80 tick species, including almost all tick genera found in China. Adult or immatures of these ticks utilize Artiodactyla as their main source of blood, and many of them also parasitize other mammals, birds, and reptiles. In contrast, only two species, *Haemaphysalis kitaokai* and *Hyalomma isaaci* feed on Perissodactyla, the former is also found on Artiodactyla, and the host range of another one also comprises Artiodactyla and Lagomorpha. Ticks feeding on Carnivora are not specific parasites, which can be found among other animals, and most of them are of *Ixodes* and *Haemaphysalis* genera. Similarly, associations of ticks and mammals of Lagomorpha, Pholidota, Primates, and Rodentia are not strictly specific. However, only two *Amblyomma* species parasitize Pholidota, and reports of *Anomalohimalaya* species come from Rodentia. Fifteen tick species of six genera can attack humans in the natural environment, but humans are not their specific hosts. Four tick species, *Ixodes vespertilionis*, *Ixodes simplex*, *Argas sinensis*, and *Argas vespertilionis* parasitize bats exclusively. Overall, only a few ticks in China have strict host specificity, and most ticks can live on different hosts. These observations are consistent with previous results that analyze host specificity of ticks (Cumming, 1998; Hoogstraal & Aeschlimann, 1982; Klompen et al., 1996; Nava & Guglielmone, 2013).

Tick–host specificity is dependent on the life cycle of ticks, as adult parasitize a group of hosts, while immatures feed on different host groups. These examples include some species of *Ixodes*, *Haemaphysalis*, *Dermacentor*, *Hyalomma*, and *Rhipicephalus* genera, and most of the *Dermacentor* species accord with this pattern. These observations strongly suggest that some ticks have host preferences during different stages of their life cycle. Another possibility is that adult and immature ticks occupy different habitats, where they can utilize different hosts (Hoogstraal & Aeschlimann, 1982).

## 6 TICK DYNAMICS UNDER ENVIRONMENTAL CHANGES AND CONTROL

As mentioned above, ticks spend most of their life cycle off hosts, exposed to environmental conditions that are largely altered by human activity and climate change. In a dynamic environment, the tick distribution and abundance and tick‐borne disease risk will be strongly influenced. For example, Gray et al. (2009) reviewed the effects of climate change on several tick species in Europe. Robinson et al. (2015) revealed that both forested habitat and temperature were important drivers of LD spread in Minnesota, USA. In recent years, China's urbanization process is accelerating (M. Chen, Lu, & Zha, 2010), and industrial structures of agriculture and animal husbandry have been largely adjusted. These will cause changes in the suitable environment and preferred hosts of ticks, and influences of these changes on tick dynamics can be expected, although there is no systematic study on this issue. In contrast, many ticks are of medical and economic importance, and the prevention and control of ticks has always been received attention. In China, there are many effective control strategies for ticks, including rotational grazing (Dong et al., 2007), chemical control (Nong et al., 2013; Rajput, Hu, Chen, Arijo, & Xiao, 2006), biological control (Ren et al., 2012; Sun et al., 2013), and vaccine development (T. T. Zhang et al., 2017). In such cases, the dynamic changes in ticks also deserve further study.

## 7 CONCLUSION AND PERSPECTIVE

Geographical distribution, host diversity, and specificity of 125 tick species in China are reviewed in this study. The tick distributions varied across different regions, Xinjiang has the most tick species records, while few species are present in some provinces. Endemic and universal species are also observed. The distribution pattern is partly due to the different ecological environments in the distributed area. Ticks feed on mammals, birds, and reptiles, most species choose mammals as their hosts. Host diversity of soft ticks is lower than that of hard ticks. Furthermore, only a few ticks have strict host specificity, and most ticks can live on different hosts. These observations are helpful for research on tick ecology and biology. However, more efforts are needed to effectively control ticks and tick‐borne diseases, including investigation of tick performance on different hosts, tick population dynamics and outbreak risk, mechanisms of tick biology and tick‐pathogen interactions, and tick control strategies.

## CONFLICT OF INTERESTS

The authors declare that there are no conflict of interests.

## AUTHOR CONTRIBUTIONS

Y.‐K. Z and X.‐Y. Z collected the data. Y.‐K. Z and J.‐Z. L. wrote the article.
